# Antifungal Effects of Saponin Extract from Rhizomes of* Dioscorea panthaica* Prain et Burk against* Candida albicans*

**DOI:** 10.1155/2018/6095307

**Published:** 2018-04-29

**Authors:** Longfei Yang, Xin Liu, Xinming Zhuang, Xuechao Feng, Lili Zhong, Tonghui Ma

**Affiliations:** ^1^Jilin Provincial Key Laboratory on Molecular and Chemical Genetics, The Second Hospital of Jilin University, Changchun 130041, China; ^2^Eye Center, The Second Hospital of Jilin University, Changchun 130041, China; ^3^Department of Spinal Surgery, The First Hospital of Jilin University, Changchun 130021, China; ^4^College of Life Science, Northeast Normal University, Changchun 130024, China

## Abstract

*Candida albicans* is the most common fungal pathogen causing serious diseases, while there are only a paucity of antifungal drugs. Therefore, the present study was performed to investigate the antifungal effects of saponin extract from rhizomes of* Dioscorea panthaica *Prain et Burk (Huangshanyao Saponin extract, HSE) against* C. albicans*. HSE inhibits the planktonic growth and biofilm formation and development of* C. albicans*. 16–64 *μ*g/mL of HSE could inhibit adhesion to polystyrene surfaces, transition from yeast to filamentous growth, and production of secreted phospholipase and could also induce endogenous reactive oxygen species (ROS) production and disrupt cell membrane in planktonic cells. Inhibitory activities against extracellular exopolysaccharide (EPS) production and ROS production in preformed biofilms could be inhibited by 64–256 *μ*g/mL of HSE. Cytotoxicity against human Chang's liver cells is low, with a half maximal inhibitory concentration (IC_50_) of about 256 *μ*g/mL. In sum, our study suggested that HSE might be used as a potential antifungal therapeutic against* C. albicans*.

## 1. Introduction

*Candida albicans* is the most common pathogenic fungus in human and could cause a series of skin and superficial mucosal tissue infections, including oral thrush and vaginitis, as well as the lethal invasive systemic candidiasis [1]. Recent years have witnessed the increase in the morbidity and mortality of* C. albicans* infections, due to the increased use of immune-suppression therapies (resulting from cancer therapies and organ transplantation), the rise in acquired immune deficiency syndrome (AIDS) patients, and the emergence of drug resistance [2, 3]. Among the nosocomial bloodstream infections,* C. albicans* is the fourth most common pathogenic agent [4].

Among the many virulence factors, the capacity of* C. albicans* to switch from yeast form to hyphal form and to form biofilms on abiotic and biotic surfaces plays a critical role in the pathogenesis [5, 6]. The hyphae of* C. albicans* could secrete Candidalysin which could damage the epithelial cells and facilitate the survival in and the escape from the macrophages [7, 8].* C. albicans* biofilms are complex structures consisted of different types of cells (yeast, hyphal, and pseudo-hyphal forms) encased by the extracellular exopolysaccharide (EPS) generated by the cells within biofilms [9]. The cells in biofilm are much more resistant to antifungal therapies compared to their planktonic counterparts [9]. The biofilm growth is responsible for the majority of* C. albicans* infections, especially those associated with medical devices such as catheters, pacemakers, dentures, and prosthetic joints [9, 10]. This fungal pathogen causes a large burden on social economy and public health, while there is a dearth of antifungal drugs. The development of drug resistance is making this worse. Therefore, developing novel antifungal agents, especially those effective against biofilm, is a pressing mission.

Natural products have been considered as a huge reservoir for developing new antifungal drugs [11, 12]. Huangshanyao (Chinese name) saponin extract (HSE) is the saponin extract from rhizomes of* Dioscorea panthaica* Prain et Burk, a traditional medicinal herb that is grown in Southwest part of China such as Yunnan, Sichuan, Guizhou, and Hunan and could be employed to treat bone injuries, hypertension, and gastric disorders [13, 14]. Belonging to Dioscoreaceae family, Huangshanyao is a perennial herbaceous twining vine. This herb is an endemic plant in China and the dried rhizomes are employed for medicinal use. Other plants under the same* Dioscoreae *genus, such as* Dioscoreae nipponica*,* Dioscoreae hypoglauca*,* Dioscoreae spongiosa, and Dioscoreae opposite, *have also been used as medicinal herbs [15]. There is also documentation about the protecting effects of this herb on cardiovascular disorders and this herb is the major and essential component of the marketed drug Diao Xin Xue Kang which has been approved by China Food and Drug Administration (CFDA) for ameliorating the symptoms of cardiovascular diseases [13]. The saponin extract (rich in over 30 steroidal saponins) is the major active components of Huangshanyao and has demonstrated activities against oxidative stress, cancers, rheumatism, and injuries induced by reperfusion [13, 14, 16]. This traditional herb also could be used as an external drug to treat infectious diseases caused by microbial pathogens such as* Lymphatic tuberculosis* and* Bacillus anthracis* [16, 17].

The present study explores the antifungal activity of HSE against* C. albicans*, as well as the inhibitory effect on the* C. albicans* biofilms. The influence of HSE on the virulent factors was also evaluated.

## 2. Materials and Methods

### 2.1. Extracts Preparation and Chemicals

The powdered saponins extract from the roots of* Dioscorea panthaica* Prain et Burk (Huangshanyao saponins extract, HSE) was provided and authorized by National Institute for Food and Drug Control of China (NIFDC). The lot number of the HSE powder is 110891-200001. For biological tests, HSE was solubilized in DMSO and stored at −20°C until use.

The standard reference compound pseudoprotodioscin (PPD) for* Dioscorea panthaica* Prain et Burk was also obtained from NIFDC. 3-(4,5-Dimethylthiazol-2yl)-2,5-diphenyl-2H-tetrazolium bromide (MTT), menadione, 2,3-bis(2-methoxy-4-nitro-5-sulfophenyl)-2H-tetrazolium-5-carboxanilide (XTT), propidium iodide (PI), and 2′,7′-dichlorofluorescein diacetate (DCFH-DA), as well as acetonitrile for high-performance liquid chromatography (HPLC) analysis, were bought from Sigma-Aldrich (Shanghai, China).

### 2.2. HPLC Analysis

HPLC analyses were performed on a Waters 2695 separation module (Waters Company, USA) equipped with a Waters 2996 photo-diode array (PDA) detector. HSE and standard compound PPD were dissolved in 50% acetonitrile. After centrifugation at 12,000 rpm for 15 minutes, supernatants of both samples were transferred into sample vials and subjected to HPLC elution. HPLC separations were obtained on an Xterra MS C18 column (4.6 × 150 mm, 2.5 *μ*M, Waters) at a temperature of 30°C. The mobile phase consisted of 0.1% formic acid in acetonitrile (mobile phase A) and 0.1% formic acid in ultrapure water (mobile phase B). The program for gradient elution was set as follows: 0–30 min, 15%–60% A, with a flow rate of 0.2 mL per minute. The volume for each HPLC analysis was set as 10 *μ*L, while the monitoring wavelength was 220 nm.

### 2.3. Strains and Culture Conditions

*C. albicans* SC5314 was bought from China General Microbiological Culture Collection Center (CGMCC) and stored at −80°C in yeast extract-peptone-dextrose (YPD, 1% yeast extract, 2% peptone, and 2% dextrose in ddH_2_O) medium supplemented with 20% glycerol. Before assays, SC5314 were subcultured twice on YPD agars (1% yeast extract, 2% peptone, 2% dextrose, and 1.8% agar) at 35°C. Prior to each assay, a colony of* C. albicans* on YPD agar was transferred into YPD broth and incubated at 28°C overnight with a speed of 140 rpm.

### 2.4. Effect of HSE on the Planktonic Growth of* C. albicans*

The effect of HSE on the growth of* C. albicans* was assessed according to the CLSI-M27-A3 guideline [18]. Briefly,* C. albicans* SC5314 cells from overnight grown cultures in YPD broth were harvested by centrifugation and adjusted to a final concentration of 2 × 10^3^ cells/mL in RPMI-1640 medium (without sodium bicarbonate, pH 7.0). 100 *μ*L of cell suspension was added to each well of 96-well plate (Corning, USA). HSE dissolved in DMSO was added to wells to achieve a twofold series of concentrations of HSE, ranging from 256 *μ*g/mL to 4 *μ*g/mL. Wells containing same volume of DMSO were set as negative controls, while wells treated with 4 *μ*g/mL amphotericin B served as positive controls. Wells containing only medium were served as blank controls. After incubation at 35°C for 24 h, the inhibitory effect of HSE was assessed by visual inspection. The MIC was defined as the lowest concentration at which no visual growth of* C. albicans* was observed.

To quantify the antifungal effect of HSE, MTT assay was performed as previously described [19]. Briefly, after visual inspection, 10 *μ*L of sterile MTT solution (5 mg/mL in PBS) was added to each well of microplates, followed by another incubation at 35°C in dark for 4 h. Then, the supernatant in each well was discarded and 100 *μ*L DMSO was added to each well to dissolve the water-insoluble formazan. A microplate reader (VarioSkan, Thermo, Germany) was used to detect the optical density (OD) at 570 nm. Viability of fungal cells in each well was calculated as viability = (OD_570  treatment_ − OD_570  blank_)/(OD_570  control_ − OD_570  blank_). These assays were performed three times.

### 2.5. Determination of the Minimum Fungicidal Concentration of HSE

The minimum fungicidal concentration (MFC) of HSE was assessed as previously described [20]. At the end of 24 h incubation at 35°C, an aliquot of 10 *μ*L from each well was taken, diluted serially, and plated on YPD agar. After incubation at 35°C for 48 h, the colonies grown on YPD agar were counted. The lowest concentration at which no colony of* C. albicans* grown on YPD agars was observed was defined as the MFC. These assays were performed for three times.

### 2.6. Time-Kill Curve Assay

To perform this assay, overnight grown* C. albicans* cultures in YPD broth were concentrated and diluted to a concentration of 10^6^ cells/mL in RPMI-1640 medium. Cell suspensions supplemented with different concentrations of HSE were incubated at 28°C with shaking (140 rpm). At indicated time points, aliquots from each culture were withdrawn, serially diluted, and spotted onto YPD agars. After incubation at 37°C for 48 h, colony forming units (CFU) on each agar plate were determined. Three independent tests were performed [21].

### 2.7. The Effect of HSE on the Adhesion of* C. albicans* to Polystyrene Surface

The impact of HSE on the adhesion of* C. albicans* to the surface of polystyrene materials was assessed by the XTT reduction assay [22]. In brief, overnight cultured SC5314 cells were resuspended in 1640 medium to obtain a concentration of 10^6^ cells/mL. 100 *μ*L of that suspension was transferred into each well of 96-well plate and treated with different concentrations of HSE (0, 16, 32, and 64 *μ*g/mL). After being incubated at 37°C for 90 minutes, wells were washed with sterile PBS for three times to remove nonadherent cells. XTT assay was carried out to determine the percentage of adherent cells of treatment wells comparing with negative controls. This assay was performed in triplicate and repeated for three times.

### 2.8. The Effect of HSE on Morphological Transition of* C. albicans*

This assay was performed as described previously [22].* C. albicans* cells at a density of 1 × 10^6^ cells/mL in 1640 medium were incubated with different concentrations of HSE at 37°C. At indicated time points (3 h, 6 h, 12 h, and 24 h) after cells were added to 96-well plate, photos of biofilms treated with different concentrations of HSE were acquired by an inverted microscope (Olympus IX71, Japan). In addition, spider agar (1% mannitol, 1% nutrient broth, 0.2% K_2_HPO_4_, 1.8% agar, and pH 7.2) with different concentrations of HSE were prepared before smearing about 50 cells (in 100 *μ*L 1640 medium) onto the solid agar to detect the effect of HSE on the morphological transition on solid agar. After 120 h incubation at 37°C, the morphologies of colonies were recorded by a stereomicroscope (Olympus SZX-16, Japan). These assays were performed in triplicate.

### 2.9. The Effect of HSE on the Formation of* C. albicans* Biofilm

The inhibitory effect of HSE on* C. albicans* biofilm formation was assessed by XTT reduction assay [23]. Overnight grown cultures of* C. albicans* SC5314 in YPD medium were collected by centrifugation and resuspended in 1640 medium to get a concentration of 10^6^ cells/mL. 100 *μ*L cell suspension containing different concentrations of HSE was added to each well of 96-well plate followed by sealing the plate with Parafilm. After 24 h incubation at 37°C, each well was washed with PBS for three times to remove the nonadherent cells. Then 100 *μ*L sterile XTT solution was added to each well followed by another 2 h incubation. 75 *μ*L supernatant from each well was transferred into fresh wells and the optical density at 490 nm was read with a VarioSkan multifunctional plate reader (Thermo, Germany). The assays were performed in triplicate and repeated for three times.

### 2.10. The Effects of HSE on the Preformed* C. albicans* Biofilm

The activity of HSE on the preformed biofilm was assessed with the same method as mentioned above [23]. After 24 h incubation without drugs, the 96-well plate containing biofilms was washed with PBS for three times to remove free-floating cells. Then fresh 1640 medium containing different concentrations of HSE was added to each well. After another 24 h incubation at 37°C, wells containing treated or nontreated biofilms were washed with PBS and XTT reduction assay was performed. The MIC for biofilm formation (MIC_biofilm  formation_) and preformed biofilm (MIC_preformed  biofilm_) were defined as the lowest concentration that inhibited more than 80% of the metabolic activity comparable to drug-free control biofilms. This assay was performed in triplicate and repeated for three times.

### 2.11. Analysis of* C. albicans* Biofilm Formation with Confocal Laser Scanning Microscope

*C. albicans* biofilms formed in 96-well plates at 37°C with different concentrations of HSE were washed with sterile PBS. The biofilms were incubated with 5 *μ*M Syto® 9 (Molecular Probes, USA) for 10 minutes which could stain all the cells and fluorescence green, irrespective of cell viability. After being washed with PBS, biofilms were photographed by confocal laser scanning microscope (CLSM) (Olympus Fluoview FV1000, Japan) with an adjusted 40x objective lens (Olympus LCAch N, UIS2, Japan). The detailed 3D images of* C. albicans* biofilms were acquired using *z*-axis scanning (step size = 2 *μ*m and the number of photo-slices is dependent on the height of biofilms). The three-dimensional images were reconstructed with Imaris 7.2.3 (Bitplane, Switzerland) to visualize the 3D structures of* C. albicans* biofilms.

### 2.12. The Effects of HSE on the Cell Membrane Integrity of* C. albicans*

The effect of HSE on the integrity of* Candida* cell membrane was evaluated by staining cell with fluorescent dye PI, which could only bind to DNA of dead cells because the integral membrane of live cells could prevent the access into cells. Briefly,* Candida* cells treated with different concentrations of HSE were incubated with 10 *μ*M PI for half an hour in the dark at 37°C. After washing with PBS for twice, images of* C. albicans* cells were acquired by CLSM.

For quantitative analysis, following treatment and incubation with PI, cells were subjected to flow cytometry (FCM) (Beckman Coulter EPICS XL-MCL, USA) equipped with an argon laser (488 nm) for excitation to detect the fluorescent intensity of cells [24]. The percentage of PI stained cells was analyzed by Expo32 ADC analysis software (Beckman Coulter EPICS XL, USA). These assays were performed in triplicate and repeated for three times.

### 2.13. The Effects of HSE on the Endogenous Production of ROS

The capacity of HSE to induce endogenous reactive oxygen species (ROS) in* C. albicans* cells was investigated using the fluorescent probe DCFH-DA [25].* C. albicans* suspensions at a density of 1 × 10^6^ cells/mL from overnight cultures were exposed to different concentration of HSE for 2 h before incubation with 10 *μ*M DCFH-DA for 20 minutes. After washing with PBS for twice, samples were subjected to flow cytometry for ROS detection.

As for ROS production in mature biofilms in 96-well plate, DCFH-DA was added to wells with biofilms to achieve a concentration of 10 *μ*M. After incubation for 20 minutes in dark at 37°C and removing excessive extracellular dye by washing, biofilms were incubated with indicated concentrations of HSE for 2 h in dark. The fluorescent intensity of biofilms was detected by VarioSkan multifunctional microplate reader at Ex 485 nm/Em 525 nm [24].

To further probe the influence of ROS on the antibiofilm activity of HSE, the metabolic viability of biofilms treated with HSE in the presence and absence of ROS inhibitor N-acetyl-cysteine (NAC) was evaluated by XTT reduction assay as aforementioned. Before the viability assay, the morphologies of biofilms were photographed with microscope. The concentration of NAC used was 150 *μ*g/mL, according to the previous study performed by others [24]. These assays were performed in triplicate and repeated at least three times.

### 2.14. The Effects of HSE on the EPS Production of* C. albicans*

The EPS production of preformed* C. albicans* biofilms was evaluated by concentrated H_2_SO_4_-phenol method as previously described [26] with small modifications.* C. albicans* biofilms cultured under the same conditions as mentioned above for 24 h in 24-well plate were washed with PBS and fresh medium with different concentrations of HSE was added to each well. Following another 24 h incubation at 37°C, the supernatant was removed from each well and 200 *μ*L 0.9% NaCl was added. An aliquot of 200 *μ*L 0.5% phenol was added to each well prior to the gentle addition of 2 mL 0.2% hydrazine sulfate (w/v, in concentrated H_2_SO_4_). After incubation in dark for 1 h, the OD_490_ nm of each sample was read by a VarioSkan multifunctional plate reader. Assays were performed in triplicate and repeated three times.

### 2.15. The Effect of HSE on the Production of Phospholipase

The production of phospholipase was evaluated by egg yolk agar method [26]. In brief, different concentrations of HSE in autoclaved egg yolk agar medium (NaCl 5.73%, 3% glucose, 1% peptone, 0.055% CaCl_2_, 10% egg yolk emulsion, 1.8% agar) were achieved as follows: after aseptic egg yolk emulsion was added to the autoclaved medium, and HSE stock solution was added to the medium and mixed well. 1 *μ*L of overnight grown* C. albicans* culture (adjusted to 10^7^ cells/mL) was transferred onto the agar. After 4 days' incubation at 37°C, for each colony, the diameter of colony and the diameter of colony plus the surrounding precipitation zone were determined. The precipitation zone (Pz) values were calculated as the ratio of the diameter of colony divided by that of the precipitation zones around the colony. The Pz values were employed to assess the activity of phospholipase as follows: Pz = 1, negative activity; Pz = 0.64~0.99, positive activity; and Pz < 0.64, very strong activity [27].

### 2.16. Cytotoxicity against Mammal Cells

MTT reduction assay was performed to assay the cytotoxicity of HSE to human Chang's liver cells [22]. Cell suspensions in DMEM medium (Gibco, China) plus 10% heat-inactivated fetal bovine serum (FBS) were seeded at 2 × 10^5^ cells/mL into 96-well plates. After 24 h incubation with or without different concentrations of HSE at 37°C in a 5% CO_2_ incubator, 10 *μ*L sterile MTT solution (5 mg/mL in PBS) was added to each well followed by another incubation of 4 h at 37°C in dark. The formazan produced by cells of each well was dissolved in DMSO after the liquid of each well was removed and the OD values at 570 nm of each well were detected by a multifunctional plate reader. The experiments were performed in triplicate and repeated three times.

### 2.17. Statistical Analysis

Data were analyzed and graphs were generated using GraphPad Prism 6.02 (GraphPad Software, USA). Statistical significance between treated and control groups was analyzed by Student's *t*-test with two-tailed and equal variance. Differences were considered statistically significant if *p* < 0.05.

## 3. Results

### 3.1. HPLC Analyses

**Figure 1:**
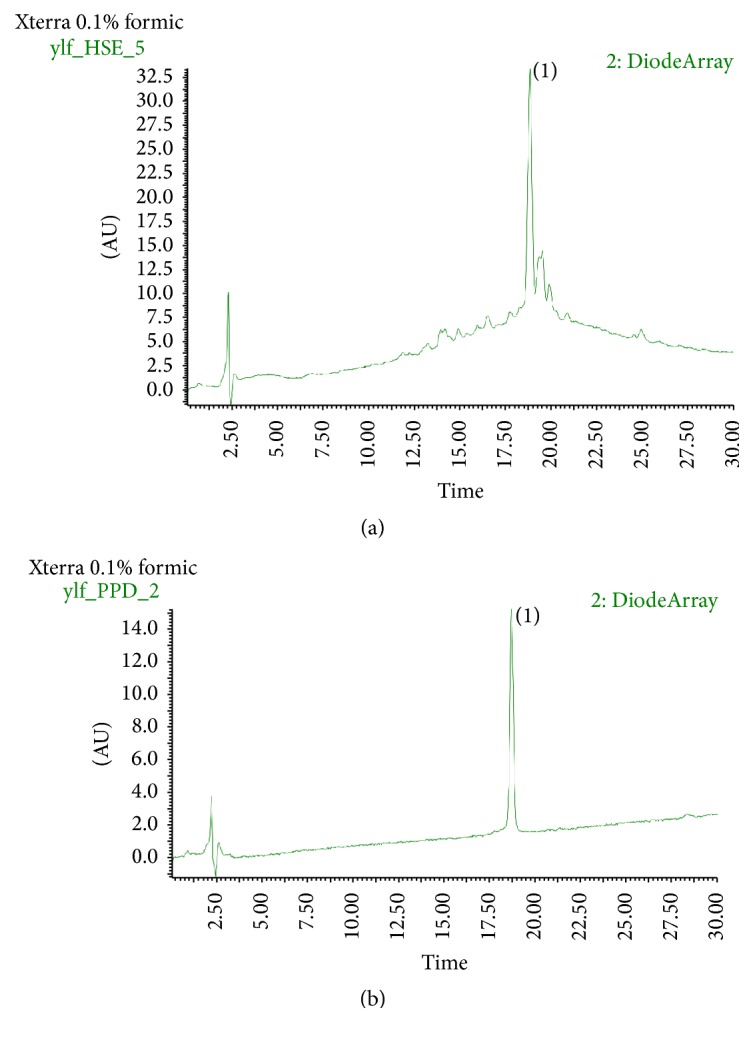
HPLC profiles at 220 nm of HSE and pseudoprotodioscin (PPD). (a) HSE, (b) PPD. Peak (1) is PPD with a retention time of 18.73 minutes. Separation was performed on a Waters 2695 equipped with a 2996 photo-diode array (PDA) detector. Mobile phase: (A) 0.1% formic acid in acetonitrile and (b) 0.1% formic acid in water. Flow rate: 0.2 mL/min. Column temperature: 30°C. Elution program: 0–30 min, 15%–60% A.

HPLC analyses were performed on a Waters 2695 separation module equipped with a 2996 detector, using acetonitrile (containing 0.1% formic acid) and ultrapure water (containing 0.1% formic acid) as mobile phases. HPLC spectrums were shown in Figure 1. PPD in this elution program has a retention time of 18.73 minutes.

### 3.2. Antifungal Susceptibility

**Figure 2:**
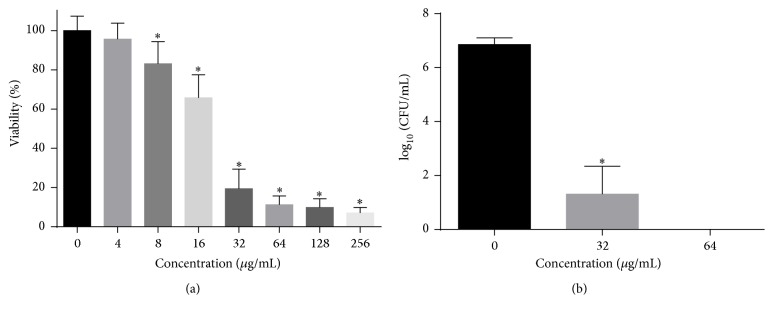
The antifungal activity of HSE on planktonic* C. albicans* cells. (a) MIC of HSE against* C. albicans* was determined by microdilution method.* C. albicans* cells were incubated with various concentrations of HSE for 24 h before MTT assay was performed. (b) MFC of HSE against* C. albicans* was obtained by counting the colonies grown on SD agars after serial dilution of each well and incubation at 37°C for 48 h. Data in the figures are average results from three independent tests. *∗* means *p* < 0.05. CFU, colony forming units.

We performed antifungal tests according to CLSI-M27-A3 guidelines. As shown in Figure 2, the MIC value of HSE against* C. albicans* SC5314 was 32 *μ*g/mL, while the MFC of HSE was 64 *μ*g/mL. The MFC/MIC value was 2, indicating the effect of HSE on* C. albicans* was fungicidal.

**Figure 3:**
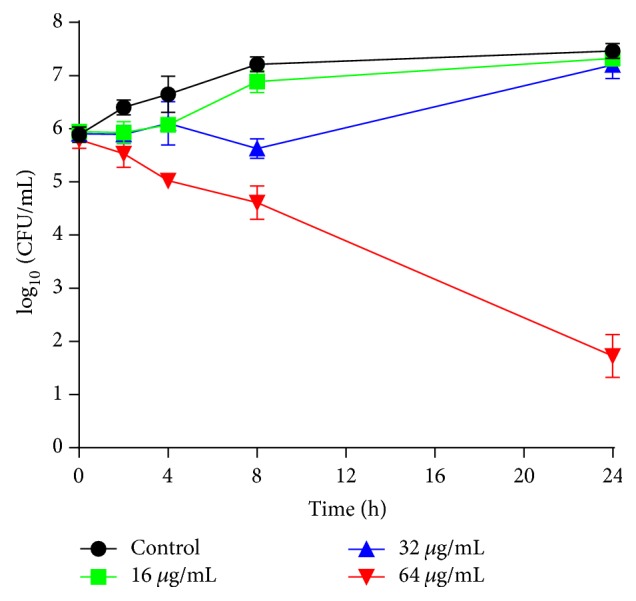
Time-kill curves of HSE against* C. albicans* SC5314.* C. albicans* suspension (10^6^ cells/mL in RPMI-1640 medium) from overnight grown cultures was incubated with different concentrations of HSE at 28°C, 200 rpm. Cell suspension with the same volume DMSO was set as control. Data presented were means ± standard deviations from three independent tests.

After the antifungal susceptibility, we performed time-kill assay. As shown in Figure 3, weak fungicidal effect was found. 16 *μ*g/mL of HSE could only slow down the growth of* C. albicans,* while 32 *μ*g/mL of HSE exerted fungicidal effect in the first 8 h before cells grown to almost 10^7^ CFU/mL, similar to that of HSE-free controls. Treatment with 64 *μ*g/mL HSE for 24 h attained strong fungicidal effect, yielding an about 4 log_10_ CFU/mL decrease compared to the beginning inoculum of the assay, which further corroborated the fungicidal effect of HSE, because the fungicidal effect could also be defined as a reduction more than 3 log_10_ CFU/mL from the initial inoculum in the time-kill assay [28].

### 3.3. The Effects of HSE on Adhesion

**Figure 4:**
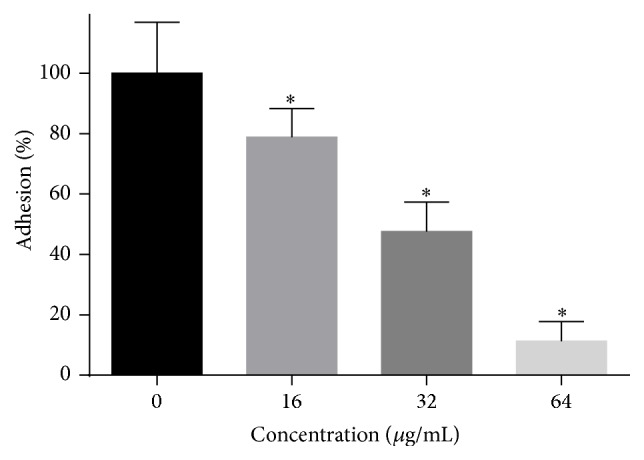
The effect of HSE on the adhesion of* C. albicans* to polystyrene plates.* C. albicans* SC5314 in 1640 medium with certain concentrations of HSE were added to 96-well plate and incubated at 37°C for 1.5 h, followed by an XTT assay to assess the adhesion rate of the cells compared to the control group. Data are shown as means + SDs, while *∗* means *p* < 0.05.

Both infections and biofilm formation of* C. albicans* start from adhesion. Therefore, we examined the influence of HSE on the adhesion of* C. albicans* to polystyrene surfaces. The data we obtained showed that 16–64 *μ*g/mL decreased the viability of adherent cells on the polystyrene surfaces (Figure 4). Treatment with 64 *μ*g/mL of HSE inhibited approximately 80% of the adhesion as compared to the drug-free control groups. These data demonstrated that HSE could suppress the adhesion of* C. albicans* to polystyrene surfaces.

### 3.4. Antibiofilm Activity

**Figure 5:**
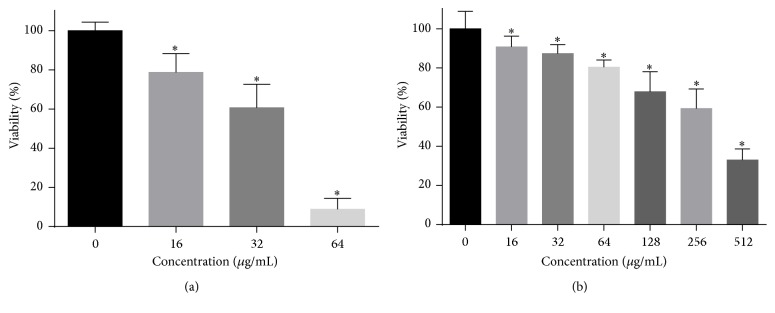
The antifungal activity of HSE on the* C. albicans* biofilm formation (a) and development (b). (a)* C. albicans* cells in 96-well plates were grown in the presence or absence of HSE at 37°C for 24 h and the metabolic activity of biofilms in each well was determined using XTT assay. (b) Twenty-four-hour mature* C. albicans* biofilms were exposed to various concentrations of HSE for another 24 h and the metabolic activities were compared to those of drug-free biofilms by an XTT reduction assay (^*∗*^*p* < 0.05).

The antibiofilm activity of HSE on* C. albicans* was evaluated by XTT reduction assay. Both the formation and development of* C. albicans* biofilms could be inhibited by HSE. As shown in Figure 5, 16–64 *μ*g/mL HSE could inhibit about 30%–90% of biofilm formation as compared to those of drug-free controls. However, until the concentration as high as 256 *μ*g/mL could HSE inhibit approximately 40% viability of preformed* Candida *biofilms.

### 3.5. The Effects of HSE on Hyphal Growth

**Figure 6:**
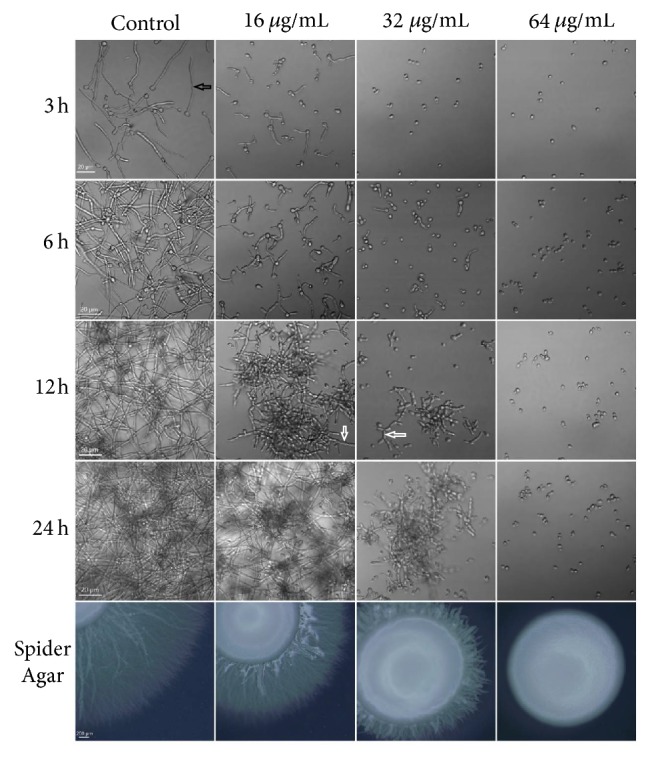
The effect of HSE on the morphological transition of* C. albicans*.* C. albicans* SC5314 cells were challenged with different concentrations of HSE under hyphae-inducing conditions (at 37°C, in RPMI-1640 medium or on spider agar). The images were acquired by Olympus microscopes at indicated times. White arrows indicate pseudohyphae, while the black arrow indicates a hypha.

The transition from yeast to hyphae plays an important role in the pathogenesis of* C. albicans* infections and is considered as the most famous virulent trait of* C. albicans*. Therefore, we tested the effects of HSE on the yeast-to-hyphal transition. In the liquid RPMI-1640 medium and solid spider agar medium, 16–64 *μ*g/mL markedly inhibited the transition in dose-dependent and time-dependent manner as demonstrated in Figure 6. In the liquid medium, cells in the control group formed true hyphae in 3 h and substantial hyphal networks in 24 h. Conversely, in comparison to control group, cells treated with 16 *μ*g/mL of HSE retarded the hyphal growth as indicated by the decreased appearance of hyphae and shortened hyphal length in the first 6 h. Treatment with 32 *μ*g/mL of HSE inhibited the formation of hyphae in 24 h and 64 *μ*g/mL of HSE entirely suppressed the morphological transition of* C. albicans*. The capacity of HSE to suppress the hyphal formation was further confirmed by morphological changes in colonies on spider agar treated with different concentrations of HSE. Drug-free colonies formed radical shapes, while increased HSE concentrations resulted in smoother peripheral regions. In summary, HSE could inhibit the yeast-to-hyphal transition of* C. albicans*.

### 3.6. Confocal Laser Scanning Microscope Analysis

**Figure 7:**
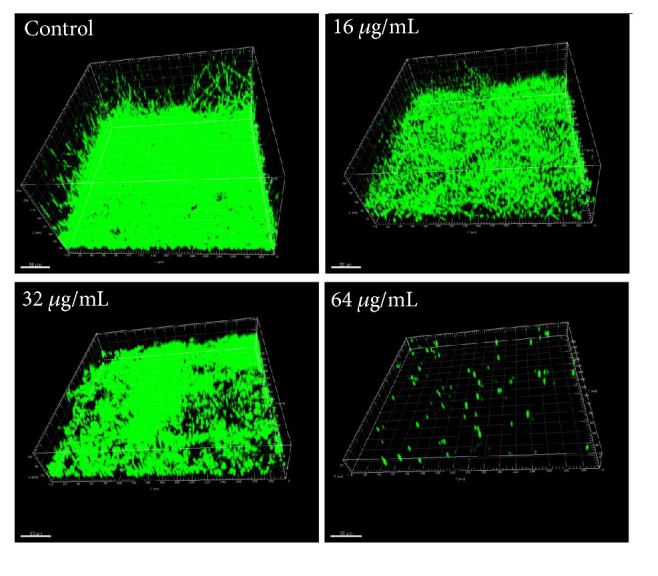
The effects of HSE on the formation of* C. albicans* biofilms. The biofilms were formed in the presence of different concentrations of HSE (0, 16, 32, and 64 *μ*g/mL) for 24 h, followed by staining with Syto 9. Pictures were taken by CLSM using the *xyz*-scanning mode and reconstructed by Imaris 7.2.3.

The effects of HSE on the 3D structures of* C. albicans* biofilms were evaluated by CLSM. As shown in Figure 7, drug-free biofilms were robust with massive long hyphae pervading in the medium, while, in contrast, incubation with 16–32 *μ*g/mL of HSE impaired the formation of biofilms significantly as indicated by the decreased heights of biofilms and the descending presence of hyphae. Moreover, 64 *μ*g/mL of HSE completely prevented biofilm formation. This was consistent with the deceased viability caused by HSE obtained through XTT reduction assay (Figure 6).

### 3.7. Cell Membrane Permeability

**Figure 8:**
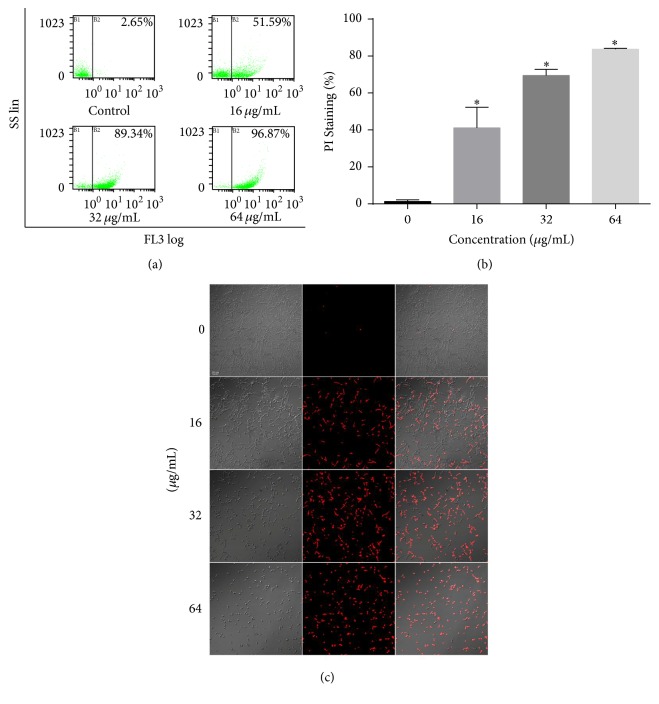
HSE treatment increases the disruption of the plasma membrane integrity. (a)* Candida* cells treated with HSE were subjected to PI staining and FCM for cell membrane integrity analysis. (b) Summary of FCM assays for PI influx. (c) Representative confocal photos of PI staining samples treated with different concentrations of HSE. ^*∗*^*p* < 0.05.

To further elucidate the possible mechanism underlying the antifungal activity of HSE, cell membrane integrity was evaluated by PI influx assay through CLSM. As demonstrated in Figure 8, HSE treatment resulted in increased PI influx in a dose-dependent way, indicating a membrane-disrupting action. To make a further step to quantify the PI influx caused by HSE, FCM was employed. Treatment with 16 *μ*g/mL HSE caused above 50% membrane disruption of the population, while treatment with 32 *μ*g/mL and 64 *μ*g/mL of HSE induced about 90% and 96% PI-positive cells in the samples, respectively, consistent with the data acquired by CLSM. In summary, HSE treatment significantly increased the membrane permeability of* C. albicans* cells.

### 3.8. ROS Production in Planktonic and Biofilm Cells

**Figure 9:**
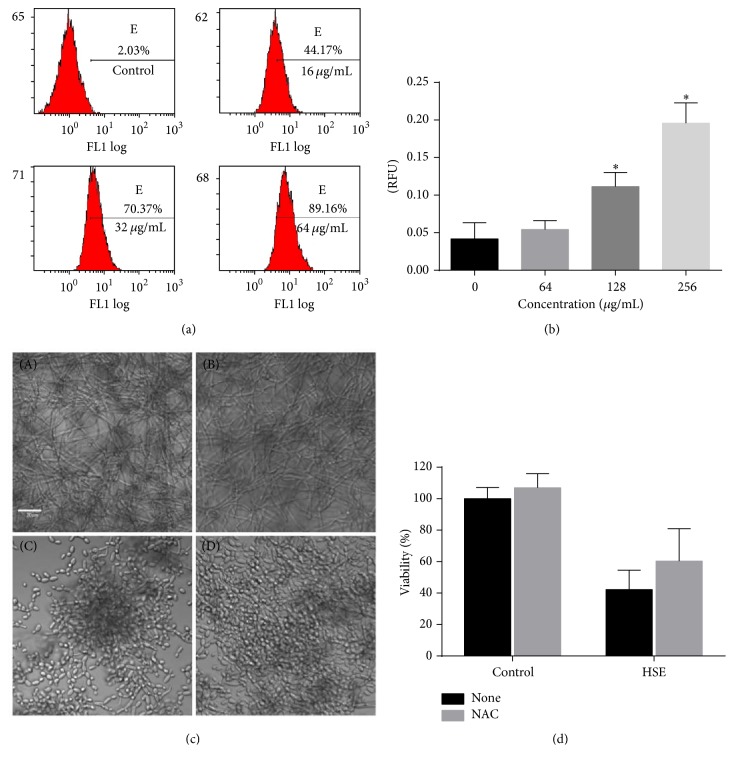
ROS involved in the antibiofilm activity of HSE. (a) HSE treatment increased the endogenous ROS production.* Candida* cells treated with different concentrations of HSE were subjected to FCM for ROS production analysis. (b) Treatment with HSE caused increased production of ROS in preformed biofilms. 24 h mature biofilms were incubated with 10 *μ*M DCFH-DA for 30 min before washing with PBS, challenging with HSE and determining fluorescence with a multifunctional plate reader at 485 nm/525 nm (Thermo VarioSkan, Germany). (c) The presence of exogenous antioxidant NAC could restore the HSE-inhibited biofilm formation. (a) Negative control (DMSO); (b) DMSO + NAC; (c) HSE; (d) HSE + NAC. The concentration of NAC is 150 *μ*g/mL, while HSE is 32 *μ*g/mL. (d) Coincubation of 150 *μ*g/mL of NAC with 32 *μ*g/mL HSE could rescue part of the biofilm viability. ^*∗*^*p* < 0.05.

Many drugs exert their antifungal effects through inducing endogenous ROS production which may impose oxidative damage to macromolecules such as DNA and proteins and finally influence the health and viability of cells [29–31]. So the accumulation of ROS was investigated by FCM using DCFH-DA staining. As shown in Figure 9, in planktonic growth, increasing the HSE concentration caused ascending ROS production. In the preformed biofilms, where the ROS production could not be detected readily by FCM method, in situ detection of ROS by microplate reader following preincubation with ROS probe was performed. As shown in Figure 9, treatment with HSE for 1 h resulted in obvious increase in the fluorescent intensity of ROS probe, indicating elevated ROS production in the mature* C. albicans* biofilms treated with HSE. To further probe the effects of ROS in the antibiofilm capacity of HSE, the antioxidant NAC was used. As shown in Figure 9, the presence of NAC rescued the viability of* C. albicans* biofilms, indicating that ROS are responsible for the antibiofilm activity of HSE.

### 3.9. The Effects of HSE on the Production of EPS

**Figure 10:**
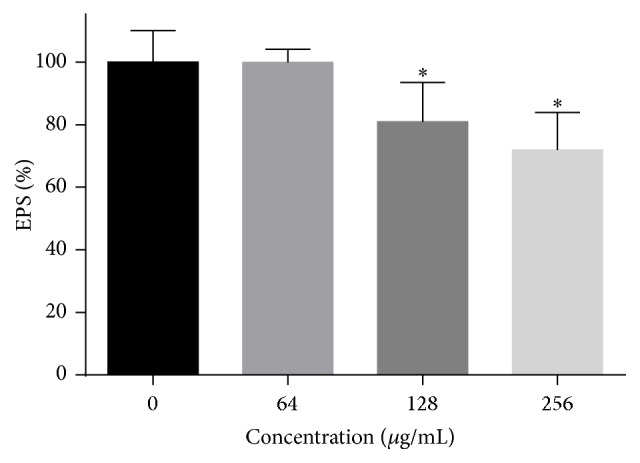
HSE treatment could decrease the EPS of preformed* C. albicans* biofilms. After preformed biofilms were treated with different concentrations of HSE for 24 h, the concentrated H_2_SO_4_-phenol method was used to determine the EPS production of biofilms. *∗* means *p* < 0.05.

Treatment with 64–256 *μ*g/mL HSE decreased the production of EPS by 25–37% (Figure 10). Since EPS contribute to the resistance of* C. albicans* to antifungal drugs and the insusceptibility to human immune system, HSE might improve this by making cells in biofilm more accessible to drugs and the components of immune systems.

### 3.10. The Effects of HSE on the Production of Phospholipase

**Figure 11:**
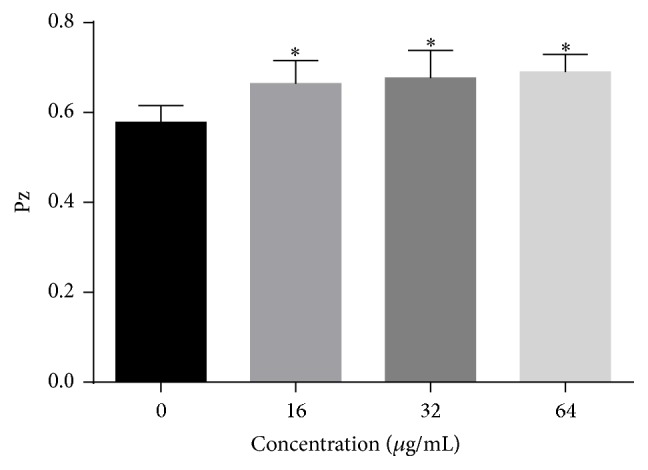
HSE decreased the production of extracellular phospholipase. Pz values were determined on egg yolk emulsion agar after incubation with or without different concentrations of HSE for 4 days at 37°C. Data shown were means + SD, while *∗* means *p* < 0.05 compared with drug-free samples.

The effects of HSE on the phospholipase production of* C. albicans* were investigated. As demonstrated in Figure 11, increasing the concentrations of HSE resulted in decreased production of phospholipase as indicated by the increasing values of Pz.

### 3.11. Cytotoxicity against Human Cells

**Figure 12:**
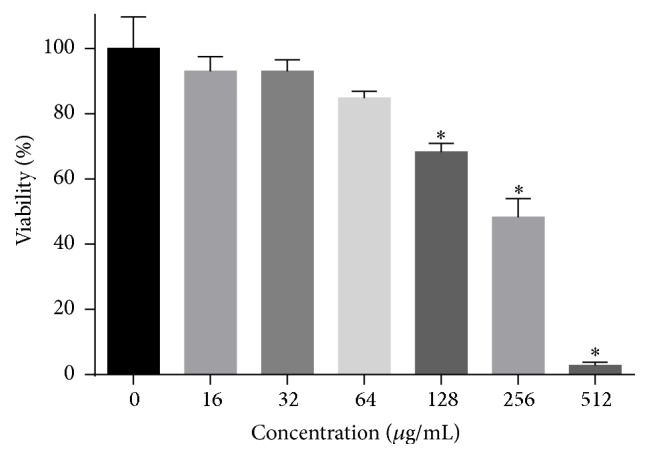
Cytotoxicity of HSE against Chang's liver cell determined by MTT assay. MTT assay was performed after treatment with different concentrations of HSE for 24 h. ^*∗*^*p* < 0.05.

In the MTT assay performed to evaluate the cytotoxicity of HSE to Chang's liver cells, HSE show low cytotoxicity with a half maximal inhibitory concentration (IC_50_) at above 200 *μ*g/mL (Figure 12) which is much higher than the MIC and MFC value of HSE against* C. albicans*. This indicates that HSE may be less toxic or nontoxic for human cells.

## 4. Discussion

The fungal pathogen* C. albicans* could impose serious health and economic burden on our society [32]. The paucity of antifungal drugs and problems with conventional therapeutics such as drug resistance, toxicity, side effects, and recurrence make developing novel antifungal agents against* C. albicans* a pressing mission [29, 33]. Various kinds of natural products with antifungal activity have been discovered [22, 34–38]. The health benefits of HSE have been shown by the marketed drug, and here, the aim of this research was to study the inhibitory effects of HSE on the proliferation, adhesion to biomaterial surfaces, morphological transition, phospholipase production, and biofilm formation of* C. albicans*.

To the best of our knowledge, this is the first research on the antifungal effects of HSE against both planktonic and biofilm types of* C. albicans*. In light of the definitions of fungicidal versus fungistatic effects [28, 34], the ratio of MFC to MIC was used to judge whether the antifungal agents had fungicidal (MFC/MIC < 4) or fungistatic (the ratio ≥ 4) effects [36]. Therefore, in our study the effect of HSE was considered as fungicidal, which was further validated by plasma integrity assay using fluorescent dye PI, because HSE could damage the cell membrane leading to PI influx.

Through the time-kill kinetics assay, the fungicidal effect of HSE was also validated according to another definition of fungicidal effects [28]. The results that 32 *μ*g/mL of HSE exhibited inhibitory effect while 64 *μ*g/mL of HSE showed strong fungicidal activity are consistent with MIC and MFC assay, although the culture conditions were different.

Adhesion, through specific cell surface adhesins, to a substrate is the first step of infection and biofilm formation. So inhibition of* C. albicans* cells adhesion could be a therapeutic target for preventing the incipient phases of* Candida* biofilm formation [39]. Consistent with many antifungal compounds exhibiting the capacity of inhibiting the adhesion of* C. albicans*, HSE also showed antiadhesion capacity [22, 34, 40, 41].

Hyphae act as an important part to the infection and biofilm formation. During the mucosal-associated infections, hyphae invade the epithelial and endothelial cells and thus introduce damage, during which the hydrolytic enzymes, such as phospholipase, play an important role [42]. The presence of hyphae makes the biofilm more cohesive, and it also helps* C. albicans* cells to cause damage to epithelial cells through candidalysin and to penetrate deeply into tissues [8]. So comes the thought that attenuating hyphal growth may possibly assuage hosts' damage [5]. Mutants incapable of forming hyphae could not induce damage to oral epithelial cells. Candidalysin secreted specifically by the hyphae of* C. albicans* also confirmed this thought indirectly [8]. During our study, HSE was able to suppress the hyphal growth of* C. albicans* both in the liquid medium and on the solid agar. Although solasodine-3-O-*β*-D-glucopyranoside could both decrease the sizes of radical periphery and smooth center on spider agars, the natural compound purpurin could only inhibit the growth of peripheral wrinkles, while the diameters of smooth centers increased as the concentration of purpurin used increased, which is consistent with our results [22, 38].

From the results we obtained, it is conjectured that HSE may function well as a good antibiofilm agent since HSE inhibits hyphal development. Therefore, we examined the effects of HSE on biofilm formation by XTT reduction assay and confocal microscopy and found that HSE did inhibit the biofilm formation. In the biofilm formation, 32 *μ*g/mL (MIC) of HSE could inhibit approximately 50% of biofilm viability, while 64 *μ*g/mL (MFC) of HSE could almost entirely prevent the biofilm formation. Combining with the time-kill kinetics, this probably indicated that the antifungal effects of HSE depended on the maturity of* C. albicans* biofilm (MIC < MIC_biofilm  formation_ < MIC_preformed  biofilm_).

As for preformed biofilms, our results showed that the concentration required for inhibiting 50% viability is much higher (approximately 8 times) than that of planktonic cells. This is the case many antifungal drugs have. The reason for increased tolerance might be that matured biofilms have accumulated extracellular matrix (predominately polysaccharides and proteins) which could prevent the access of drugs into cells within biofilms and strengthen the structures of biofilms [10, 43]. In our study, HSE could inhibit the EPS production of preformed biofilms, a characteristic of many antifungal compounds such as thiazolidinedione-8, usnic acid, and 2,4-di-tert-butylphenol [26, 35, 44].

The fluorescent dye PI has been widely used to detect the membrane permeability because this membrane impermeable probe could only enter cells with permeability-compromised membrane [24, 34, 45]. FCM and CLSM assay showed that HSE caused significant damage to the plasma membrane of* C. albicans *cells, while the membrane-perturbation effects of HSE are akin to many other antifungal agents, for example, antimicrobial peptides, diphenyl diselenide, 3,5-di-tert-butylphenol, hibicuslide C, and thymoquinone [24, 45–48].

Although antioxidant activity of steroidal saponins of* Dioscorea panthaica* Prain et Burk has been documented [13], our current results showed that saponins extract of this herb could induce endogenous ROS in both planktonic and mature biofilm cells and the presence of antioxidant NAC could increase the viability of biofilms challenged by HSE. The discrepancy might result from the differences in the objects used. These results suggested that ROS might be responsible for the antibiofilm activity of HSE. ROS can facilitate damage to DNA, proteins, and cell membranes, resulting in the cell death [49]. The ROS production in* C. albicans* cells induced by HSE was in accordance with many ROS-inducing antifungal drugs such as amphotericin B, miconazole, and caspofungin [29].

The extracellular phospholipase could hydrolyze the phospholipids in the cell membrane, thus contributing to invasion and pathogenicity [50]. Mutants defective in phospholipase showed attenuated virulence in a systematic infection model of mouse [51, 52]. Moreover, the activity of extracellular phospholipase could be predictive of mortality [53]. Our data showed HSE could suppress the secretion of phospholipase, which was consistent with other antifungal agents such as 2,4-di-tert-butylphenol and quercetin [26, 54].

Many antifungal drugs failed because of toxicity, so we evaluate the toxicity of HSE toward human hepatoma cells. According to the results we obtained, HSE showed low cytotoxicity to Chang's liver cells.

## 5. Conclusions

The present study connoted that HSE has strong fungicidal activity against planktonic* C. albicans*. HSE could also inhibit virulence factors such as adhesion, filamentous growth, biofilm formation and development, and phospholipase production. The antifungal effects might be though membrane disruption and ROS production. Considering the negligible cytotoxicity and strong antifungal activity, as well as the fact that it is the main component of the marketed drug, HSE could be thought as a promising candidate for antifungal drug development.
